# The Effect of Small Cosolutes that Mimic Molecular Crowding Conditions on the Stability of Triplexes Involving Duplex DNA

**DOI:** 10.3390/ijms17020211

**Published:** 2016-02-05

**Authors:** Anna Aviñó, Stefania Mazzini, Raimundo Gargallo, Ramon Eritja

**Affiliations:** 1 Institute for Advanced Chemistry of Catalonia (IQAC), CSIC, Jordi Girona 18-26, Barcelona 08034, Spain; aaagma@cid.csic.es; 2 Networking Center on Bioengineering, Biomaterials and Nanomedicine (CIBER-BBN), Jordi Girona 18-26, Barcelona 08034, Spain; 3 Department of Food, Environmental and Nutritional Sciences (DEFENS), Section of Chemical and Biomolecular Sciences, University of Milan, Via Celoria 2, Milan 20133, Italy; stefania.mazzini@unimi.it; 4 Department of Analytical Chemistry, University of Barcelona, Martí i Franquès, 1-11, Barcelona 08028, Spain; raimon_gargallo@ub.edu

**Keywords:** molecular crowding, triplex forming oligonucleotide (TFO), DNA, RNA, cosolute, thermal stability

## Abstract

Triplex stability is studied in crowding conditions using small cosolutes (ethanol, acetonitrile and dimethylsulfoxide) by ultraviolet (UV), circular dichroism (CD) and nuclear magnetic resonance (NMR) spectroscopies. The results indicate that the triplex is formed preferentially when the triplex forming oligonucleotide (TFO) is RNA. In addition, DNA triplexes (D:D·D) are clearly less stable in cosolute solutions while the stability of the RNA triplexes (R:D·D) is only slightly decreased. The kinetic of triplex formation with RNA-TFO is slower than with DNA-TFO and the thermal stability of the triplex is increased with the salt concentration in EtOH-water solutions. Accordingly, RNA could be considered a potential molecule to form a stable triplex for regulatory purposes in molecular crowding conditions.

## 1. Introduction

The structural polymorphism of nucleic acids is due to the inherent conformational flexibility. DNA molecules are capable of adopting a variety of non-canonical structures, including triplex, G-quadruplex or i-motif. It is becoming evident that the structural heterogeneity of nucleic acids plays an important role in relevant processes such as replication, transcription or condensation [1,2]. Pyrimidine-purine tracts, which are widespread in the eukaryotic genome, may adopt triple-stranded structures that are obtained upon binding of single-stranded DNA segments to the major groove of duplex DNA [3,4]. Triplex structures have attracted considerable interest since the discovery of intramolecular triple helices *in vivo* [5] and the possibility of targeting duplex DNA with short triplex forming oligonucleotides (TFOs) [6,7]. Therapeutic applications of TFO in the antigene strategy or in the triplex-directed modification of genes have been reported [4,8,9]. In parallel triplex motif, pyrimidine-TFOs bind to duplex via Hoogsteen hydrogen bonds forming C+:G·C and T:A·T triplets. Several factors including length, base composition, divalent cations and temperature are critical features in the formation of this structure [10].

RNA- and DNA-TFOs form stable triple helices. It has been described that the thermal stability of triplex structures in which TFO is an RNA strand (R:D·D) is higher than triplexes formed by DNA-TFOs (D:D·D) [11,12,13]. However, other authors have found similar stabilities for both triplexes [14,15].

Most of the physicochemical studies of biomolecules are performed under diluted conditions, far from the cellular environmental milieu. A characteristic of all cells is the high total concentration of macromolecules (20%–40%), they contain, leading to a crowded intracellular environment. This steric exclusion or molecular crowding generates considerable energetic and kinetic consequences. The structure and stability of biomolecules in crowding conditions remain in most of the cases unclear, and it is difficult to mimic and understand their physiology and metabolism *in vivo*. One approach to understand the forces responsible for the stability of nucleic acids mimicking cell conditions is to examine the effect of organic solvents or solutes acting as crowding and/or dehydrating agents [16,17,18].

Proteins, such as albumin or hemoglobin can be used to mimic crowded intracellular environments. However, it is difficult to analyze this effect because proteins may degrade or bind nucleic acids. Small cosolutes such as ethanol, urea or glycerol are convenient reagents for molecular crowding/dehydrating studies. These reagents do not promote an excluded volume effect but clearly alter the solution properties since they change the dielectric constant or induce an osmotic stress [16,19]. In particular, the addition of cosolutes reduces water activity and thus favors the structures or less hydrated states.

The result of adding cosolutes such as ethanol (EtOH), polyethyleneglycol (PEG), dimethylsufoxide (DMSO), or dimethylformamide (DMF) is a decreased stability of short DNA, RNA duplexes or hairpins [20,21,22]. No clear dependence between dipole moment, viscosity or surface tensions and duplex stability of the different cosolutes was observed. In addition, the tendency to destabilize the duplex structure depends on the length and the sequence composition as well as in backbone modification [23,24,25,26]. Cosolutes also have an influence on the kinetics of base pairing. For example, osmotic pressure changes may be responsible for the altered association and dissociation of DNA duplexes [25].

Quadruplex and triplex structures are recognized by regulatory proteins and often are sequence specific. These properties make them interesting for therapeutic purposes [27]. Several studies have investigated the effect of crowding/dehydrating conditions on relevant G-quadruplex structures [28,29] or G-triplexes [30]. Cosolutes have a crucial influence on the folding topology as well as on the interactions with proteins or small molecules [31]. Less attention has been paid to the effect of cosolutes on triplexes structures. Assuming a dehydrating effect of the cosolutes, the formation of the Hoogsteen base pair in a triplex is generally favored by cosolutes because it is a water releasing reaction. By contrast, the formation of the Watson–Crick duplex within the triplex is unfavored as it is considered a water-uptake reaction [32,33,34]. In addition, triplex stability is also favored in anhydrous media using deep-eutectic solvents [35].

Most of the studies on triplex stability in crowding conditions are conducted using homopolymer sequences, and in PEG solutions [32]. Here, we studied the effect of small cosolutes (EtOH, acetonitrile (ACN) and DMSO) that “mimic” molecular crowding or dehydrating agents on the formation and stability of short triplex structures. These structures are formed by short polypurine-polypyrimidine hairpins and DNA or RNA polypyrimidine triplex forming strands (Table 1). These model sequences have been used for *in situ* hybridization (triple helical COMBO-FISH, COMBinatorial Oligo Fluorescence In Situ Hybridization) as they are present in several sites of the human genome [36]. In addition, the polypurine sequence present in hairpin 1 has been previously studied as a model sequence for the design of circular TFOs with ss-DNA/RNA targets [37].

**Table 1:** Sequences of oligonucleotides used in this study. (EG)_6_ indicates the hexaethyleneglycol linker.

Oligonucleotide	Sequence (5′-3′)
Hairpin 1	AGGAAGGAAAAG-(EG)_6_-CTTTTCCTTCCT
DNA TFO 1	TCCTTCCTTTTC
RNA TFO 1	UCCUUCCUUUUC
Hairpin 2	GGAAAGGAGAAAAGA-(EG)_6_-TCTTTTCTCCTTTCC
DNA TFO 2	CCTTTCCTCTTTTCT
RNA TFO 2	CCUUUCCUCUUUUCU

## 2. Results

### 2.1. Thermal UV Experiments

**Figure 1:**
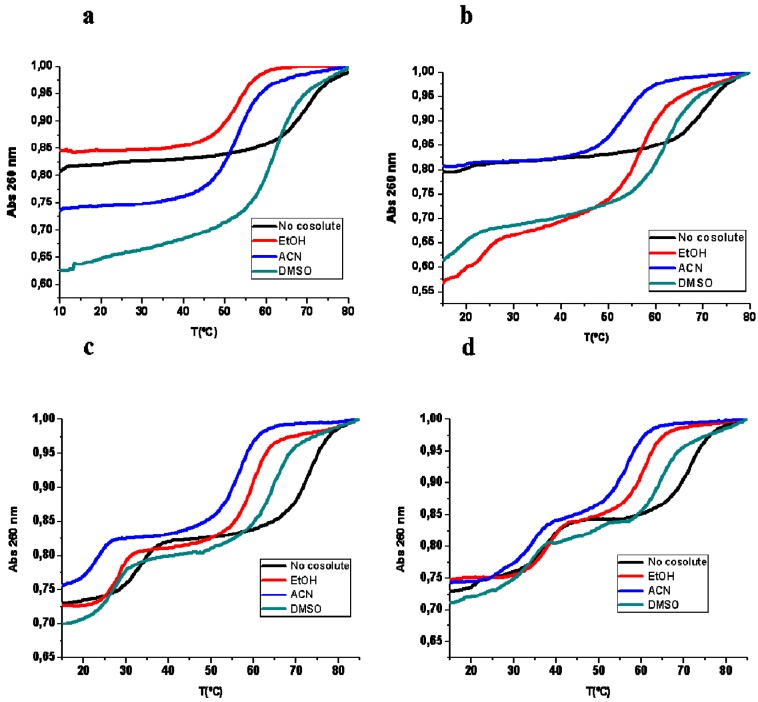
Normalized melting curves of triplexes formed by hairpin sequences with the corresponding DNA and RNA TFOs. (**a**) hairpin 1 and DNA TFO 1; (**b**) hairpin 1 and RNA TFO 1; (**c**) hairpin 2 and DNA TFO 2; (**d**) hairpin 2 and RNA TFO 2 in the presence of 20% (*v*/*w*) of cosolutes, 10 mM phosphate buffer 100 mM NaCl pH 6.

The stability of triplex structures was estimated by heating experiments recording the UV absorbance as a function of temperature. We have used hairpins instead of two separated strands to form the duplex Watson–Crick to increase the melting temperature of the duplex. Then, the association of TFO to duplex, which is normally less stable than duplex, can be studied separately. The stabilities of the triplexes formed with hairpin 2 and TFOs 2 are higher than triplexes of sequence 1 as expected. The addition of cosolutes clearly modifies the thermodynamic transitions of the triplex and duplex (Figure 1).

In the absence of cosolutes, TFO DNA 1 and TFO RNA 1 form triplex structures with hairpin 1 that have a melting temperature of 16.0 and 17.9 °C, respectively at pH 6 (Table 2). The hyperchromicity is low, between 2% and 3% indicating the formation of a small amount of triplex due to the short length of the polypurine-polypyrimidine sequence. The melting temperature of the hairpin 1 is 67.6 °C. We expect the same *T*_m_, as the DNA hairpin is the same molecule in both R:D·D and D:D·D samples, but we found some differences. These differences may reflect the presence of minor species such as intermolecular duplexes.

The addition of cosolutes clearly affects the formation of triplex and hairpin structures. The stability of the hairpin is decreased as indicated by a decrease of the melting temperatures (*T*_m_) of the duplex denaturation with the addition of EtOH. In this case, we also observed a difference in the melting temperature of the DNA hairpin with TFOs. Most importantly, the triplex dissociation is clearly observed when the TFO 1 is RNA whereas the increase of absorbance associated with the triplex formed with TFO DNA 1 is very low. Specifically in the presence of EtOH (20% *w*/*v*), the denaturing transition of triplex TFO RNA 1-hairpin 1 is 22.7 °C, almost 5 °C higher than in the absence of a cosolute. The same tendency is observed with TFOs 2. In this case, triplex with TFO RNA 2 is ten degrees higher than TFO DNA 2. EtOH acts as a dehydrating cosolute, and the formation of Hoogsteen bonds that require removal of water molecules is favored in this medium.

**Table 2:** Melting temperatures (*T*_m_, °C) calculated from thermal denaturation curves of hairpin duplex and the expected triplexes formed with hairpin and DNA- or RNA-triplex forming oligonucleotides (TFO) ^a^.

*T*_m_ (°C)	Expected Transition	No Cosolute	20% EtOH	20% ACN	20% DMSO
Hairpin 1 duplex	Duplex to SS	67.6 (26)	50.6 (28)	53.6 (25)	61.8 (40)
Hairpin 1 duplex + TFO DNA 1	Triplex to Duplex	16.0 (3)	–	–	–
	Duplex to SS	67.6 (15)	55.8 (21)	52.5 (30)	61.4 (40)
Hairpin 1 duplex + TFO RNA 1	Triplex to Duplex	17.9 (2)	22.7 (17)	19.2 (2)	18.5 (11)
	Duplex to SS	69.4 (20)	56.4 (37)	52.5 (21)	60.8 (32)
Hairpin 2 duplex	Duplex to SS	63.0 (29)	54.6 (29)	54.7 (30)	63.4 (40)
Hairpin duplex 2 + TFO DNA 2	Triplex to Duplex	33.2 (11)	27.7 (11)	23.1 (10)	27.3 (12)
	Duplex to SS	72.3 (14)	59.7 (20)	56.8 (19)	64.4 (22)
Hairpin duplex 2 + TFO RNA 2	Triplex to Duplex	37.6 (13)	37.0 (14)	31.8 (13)	33.4 (9)
	Duplex to SS	71.5 (19)	61.0 (17)	56.9 (17)	64.7 (18)

^a^ 10 mM phosphate buffer 100 mM NaCl pH 6 and the 20% *w*/*v* of cosolute; Heating rate: 0.5 °C/min. Hyperchromicity (%) in parentheses; Concentration: 1.1 μM.

In ACN, triplex stability is clearly lower than in EtOH and in the absence of a cosolute when TFO DNA is used, whereas the use of TFO RNA reduces these differences. The thermal stability of the hairpin in absence or presence of TFOs is similar. Finally, using DMSO as a cosolute, a melting transition related to the triplex dissociation is similar or slightly lower than in the absence of cosolute. Additionally, the same tendency as in EtOH and in the absence of a cosolute is observed, a triplex with TFO RNA is more stable than one with TFO DNA. The duplex hairpin transition has the highest melting temperature for the hairpin in the presence of the cosolutes. In relation to hyperchromicity associated to the melting transitions, those increase in the presence of cosolutes, especially in DMSO. Hyperchromicities of triplex transition are low (2%–3%) in the absence of cosolutes or in can, but they increase to 17% or 11% in EtOH or DMSO, respectively. This indicates the formation of a higher amount of the RNA:DNA·DNA triplex in the presence of EtOH and DMSO. However, when the triplexes are more stable (as found with TFOs 2), similar hypercromicities are observed.

Melting curves were also measured at 295 nm to confirm triplex formation (Figure S1 and Table S1). At 295 nm, triplex dissociations are associated with a decrease of the UV absorption, while duplex denaturations are not clearly observed. Additionally, a strong hysteresis between dissociation and triplex formation was observed that is consistent with a bimolecular process with slow association rates (Figure S1).

Thermodynamic parameters associated to the unfolding transitions [38,39] are shown in Table 3 and Table S2. These parameters have been evaluated based on a two-state assumption, although hysteresis was observed between the melting and annealing curves. Both, hairpin 1 and hairpin 2 show the highest stability in the absence of cosolutes, in terms of ΔG^°^_37_. Whereas the presence of 20% DMSO reduces slightly its stability, a 20% ACN-containing buffer produces a clear destabilization effect. Finally, the presence of 20% EtOH produces an intermediate effect to those observed in 20% DMSO or 20% ACN-containing buffers. In relation to triplex structures, the structure with RNA-TFO appears to have a greater stability than DNA-TFO in all the considered media. In general, the stability of triplex structure shows the highest values in the absence of cosolutes, whereas the lowest values are observed in 20% ACN-containing buffers. As in the case of hairpins 1 and 2, addition of 20% DMSO and 20% EtOH in RNA triplexes produces a small destabilization in relation to the values observed in the absence of cosolutes. In all cases involving cosolute solutions, the most stable triplex is the RNA triplex in 20% ethanol (ΔG^°^_37_ −2.6 Kcal/mol for sequence 1 and −8.9 for sequence 2).

Comparing the differences in stability of the DNA-triplex and the RNA-triplex of sequence 2, it can be observed that the difference on free energy (ΔΔG^°^) without cosolutes is 2.0 Kcal/mol (ΔG^°^_37_ = −7.2 Kcal/mol for DNA-triplex 2 and −9.2 Kcal/mol for RNA-triplex 2) while this difference is higher in cosolute solutions (ΔΔG^°^ = 5.2 in EtOH, 6.9 in ACN and 3.3 in DMSO solutions) as DNA-triplexes are strongly destabilized in cosolute solutions.

**Table 3:** Apparent thermodynamic parameters (ΔG^°^_37_, Kcal/mol) calculated from thermal denaturation curves of hairpin duplex and the expected triplexes formed with hairpin and DNA- or RNA-TFOs.

ΔG^°^_37_ (Kcal/mol)	Expected Transition	No Cosolute	20% EtOH	20% ACN	20% DMSO
Hairpin 1	Duplex to SS	−6.3	−3.4	−4.0	−5.2
Hairpin 1 + TFO DNA 1	Triplex to Duplex	0.8	–	–	–
	Duplex to SS	−6.5	−4.6	−3.5	−5.0
Hairpin 1 + TFO RNA 1	Triplex to Duplex	1	−2.6	1.3	−1.5
	Duplex to SS	−6.9	−4.7	−3.4	−5.2
Hairpin 2	Duplex to SS	−5.9	−4.3	−3.7	−5.5
Hairpin 2 + TFO DNA 1	Triplex to Duplex	−7.2	−3.7	−0.6	−4.3
	Duplex to SS	−9.4	−7.8	−5.9	−8.6
Hairpin 2 + TFO RNA 1	Triplex to Duplex	−9.2	−8.9	−7.5	−7.6
	Duplex to SS	−11.2	−8.3	−6.2	−10.0

### 2.2. Circular Dichroism Experiments

**Figure 2:**
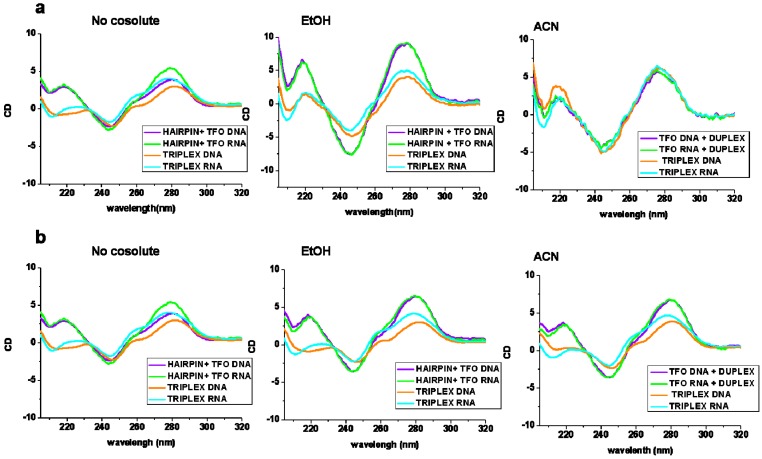
Comparison of CD spectra of the arithmetic sum of CD spectra of hairpin and TFOs (Hairpin + TFO-DNA (violet) and Hairpin + TFO-RNA (green)) and the CD spectra of the triplex structures (Triplex DNA (orange) and triplex RNA (blue)). (**a**) sequence 1 and (**b**) sequence 2. Buffer 10 mM phosphate buffer, 100 mM NaCl, pH 6.

Changes in the conformation of nucleic acids can be detected by circular dichroism (CD), so that the comparison of experimentally measured spectra with those corresponding to known structures may suggest a particular conformation. Hence, the appearance of a negative band at short-wavelength (210–220 nm) in the CD spectra indicates the formation of a parallel triplex [40,41,42]. The CD spectra of the triplex formed between the hairpin 1 and the corresponding TFOs at different cosolutes of this region are shown in Figure 2 and Figures S2 and S3 (supplementary section).

In addition, Figure 2 shows the comparison of spectra of the triplexes and the arithmetic sum of the individual CD spectra of hairpin and TFOs (individual hairpin and TFO CD spectra are shown in Figures S2 and S3). In the absence of cosolutes, the triplex formed by RNA TFO showed the strongest negative band at 210 nm. The triplex formed with DNA TFO is not very stable due to the low hyperchromicity and the low melting temperature (~16 °C) in the conditions in which the spectra were recorded. For these reasons, there are no differences between the CD spectra of the triplex sample and the arithmetic sum of individual spectra. The CD spectra of the triplex with RNA TFO and the sum of spectra of hairpin and RNA TFO are different at 210 nm confirming the formation of the triplex.

In EtOH, we observed an increase in the intense negative band (near 210 nm) upon addition of the RNA TFO to the hairpin indicating the formation of the triplex. This band also increased and became negative with the addition of DNA TFO. The formation of triplex structures is also confirmed in EtOH as the sum of the spectrum of hairpin and TFOs alone have a positive band near 210 nm, whereas the formed triplexes with both TFOs have negative bands at this wavelength (Figure 2). In ACN, the negative band at 210 nm is visible in the sample of the triplex formed with RNA TFO. The sample of triplex with DNA TFO has similar CD spectra to the CD of the hairpin indicating that the triplex is not formed in this situation. In DMSO, spectra are difficult to interpret in the 200–240 nm region owing to background noise so triplex structures in DMSO could not be confirmed by CD measurements.

### 2.3. NMR Studies

We studied the effect of ethanol and DMSO on the stability of the triplexes formed by hairpin sequence 1 at pH 6.0 by proton NMR. Imino proton spectra of the oligonucleotide hairpin 1 alone and of the triplex with TFOs with the addition of 20% of deuterated ethanol and DMSO were acquired at different temperatures.

The spectra of the hairpin in phosphate buffer and in the presence of DMSO showed well resolved signals between 12.0 and 14.5 ppm. Seven signals for the thymidine (13.5–14.5 ppm) and four signals for guanosine (12.5–13.0 ppm) were assigned. The same signals appear slightly broader in 20% of ethanol. Melting experiments showed that the presence of the cosolutes decreases the stability of the duplex in agreement with the UV data.

**Figure 3:**
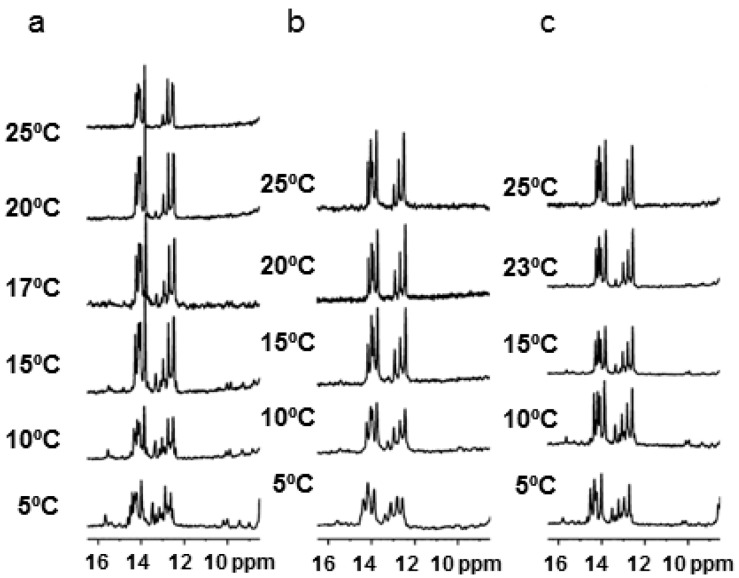
(**a**) ^1^H-NMR spectra at different temperatures of the triplexes formed by the addition TFO DNA 1 to the hairpin 1 without cosolute; (**b**) in 20% EtOH; (**c**) in 20% of DMSO. Buffer 10 mM phosphate buffer, 100 mM NaCl pH 6.0.

The imino region of ^1^H-NMR spectra of the triplex formed by the hairpin and the DNA TFO at different temperatures are shown in Figure 3. The spectra in phosphate buffer and in the presence of 20% *w*/*v* cosolutes display at 5 °C new and very broad resonance around 15.0–16.0 ppm and between 13.0 and 13.5 ppm, which are not observed in the hairpin. These findings are consistent with the formation of a triplex helix. However, all the exchangeable protons appear much narrower in the absence of cosolute or in DMSO than in the presence of ethanol, where the line broadening could be due to conformational equilibria between different species (triplex, hairpin, duplex and single strand) even at a low temperature. The melting temperature of the all-DNA triplex (Table 2) proves that the addition of cosolutes lowers the melting temperatures of the hairpin in the triplex, and this is confirmed by NMR data. In Figure 3b one may observe the disappearance of the signal at around 15 ppm related with the Hoogsteen pairing at 15 °C in ethanol and similar signals disappeared at 23 °C in DMSO. The concentrations of the triplex structures in NMR samples are higher than in UV-melting experiments. As a consequence, the triplex structure that is a bimolecular process is favored, and one may observe NMR signals corresponding to triplex at a higher temperature than UV-melting experiments.

**Figure 4:**
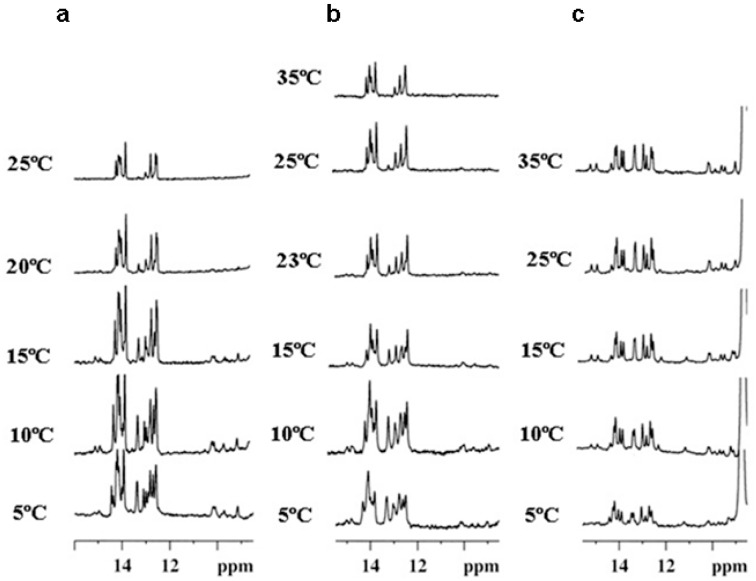
(**a**) NMR spectra at different temperatures of the triplexes formed by the addition TFO RNA 1 to the hairpin 1 without cosolute; (**b**) in 20% EtOH; (**c**) in 20% of DMSO. Buffer 10 mM phosphate buffer, 100 mM NaCl pH 6.0.

The triplex formed by the hairpin and RNA-TFO shows a different behavior in the presence of different cosolutes (Figure 4). The addition of ethanol induces a broadening of the imino signals that difficults the observation of the signals, but some triplex signals are still observable at 23 °C. In addition, the triplex transition with DMSO is difficult to assign as the signals in the region 13–15 ppm endure with the heating up to 35 °C. Nevertheless, some signals in the region 10–11 ppm disappear between 15 and 35 °C where the UV triplex melting transition was observed.

### 2.4. Kinetic Studies of Triplex Formation under Crowding Conditions

It is known that the kinetics of triplex formation is approximately 10^3^ times slower than that of duplex [43,44,45]. Specific modification of TFO, as well as the addition of cosolutes, may increase the rate of triplex formation and consequently the gene-targeting efficacy [38,46,47,48].

The mechanism of triplex formation may cause structural changes in the duplex [49] and may be explained by a nucleation-zipping model [50] in which the association rate constant is governed from the base triplets on the 5′ end of the duplex purine strand. Destabilization of the duplex by cosolutes may cause structural changes that promote the recognition and association of the TFO to the major groove.

In this work, the kinetics associated with the triplex formation in the presence and in the absence of 20% (*w*/*v*) EtOH has been studied at 15 °C. Here, absorbance decay curves in which the triplex formation is accompanied by decrease in absorbance were recorded. Next, forward (k1) and backward (k-1) rate constants were determined from the least-squares fitting of the absorbance decay curves using appropriate equations [43].

**Figure 5:**
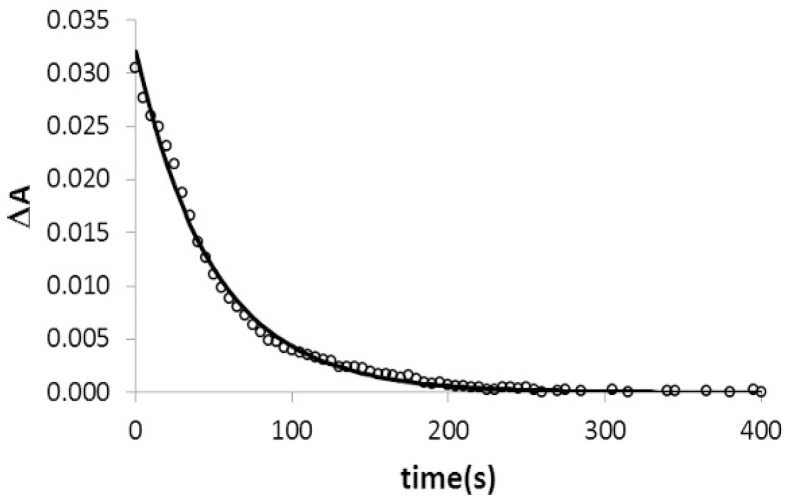
Absorbance decay recorded at 15 °C in 20% EtOH (symbols). The decay was obtained by measuring the absorbance at 270 nm as a function of time after adding to a cell containing the hairpin at 1.1 μM an equimolar amount of TFO RNA 1. The decay curve was fitted to previously developed equations [43] to determine the association and dissociation rate constants (continuous line).

For triplex formation of hairpin 1 with TFO RNA 1 in the presence of EtOH, the calculated forward and backward rate constants were 4.9 (±0.8) × 10^3^ M^−1^·s^−1^ and 1.4 (±0.3) × 10^−2^ s^−1^, respectively, according to Figure 5. These values were similar to those determined previously for a series of triplex with higher melting temperatures [43,44,45]. Finally, other rate constants could not be determined due to the low melting temperatures of the triplexes studied, which hinder the complete formation of the triplex at the end of the experiment and the subsequent corresponding mathematical analysis.

### 2.5. Effect of Cosolutes on Cation Binding to Triplex Structure

Cosolutes may change the affinity of cations for triplex structures by modifying the dielectric constant of the solution. All cosolutes that have been studied in this work have a lower dielectric constant than water, so the addition of cosolutes decreases the dielectric response and significantly influences the strength of electrostatics interactions among the triplex structure. The formation of this structure entails an increase of backbone charge density. The salt concentration dependence of a triplex stability is related to an ion binding to the strands and influences both the hairpin and the triplex stability. An increase of salt concentration favors the formation of triplex DNA [45,49] as the cations migrate to vicinity to balance phosphate groups. The addition of cosolutes changes the spatial and dielectric response of this system. Table 4 shows the stability of the triplex with an increasing concentration of salt in the presence of ethanol. Hairpin stability is highly dependent on salt concentration, but in the presence of EtOH, the changes in stability are less dramatic. Triplex stability is also improved with increasing salt concentration. At a high salt concentration, the addition of EtOH does not stabilize the triplex even with RNA-TFO.

**Table 4:** Melting temperatures (*T*_m_, °C) calculated from thermal denaturation curves of hairpin duplex and the expected triplexes formed with hairpin and DNA- or RNA-TFOs in the presence of EtOH and 10 nM–1 M NaCl solutions ^a^.

*T*_m_ (°C)	Expected Transition	10 mM NaCl No Cosolute	10 mM NaCl 20% EtOH	1000 mM NaCl No Cosolute	1000 mM NaCl 20% EtOH
Hairpin duplex 1	Duplex to SS	58.7	57.0	>80	62.1
Hairpin duplex 1 + TFO DNA 1	Triplex to Duplex	–	–	23	–
	Duplex to SS	58.6	57.5	>80	61.8
Hairpin duplex 1 + TFO RNA 1	Triplex to Duplex	–	–	23	18.0
	Duplex to SS	57.5	54	>80	61.6

^a^ 10 mM phosphate buffer and the indicated concentration of NaCl pH 6 and the same buffer with 20% *w*/*v* of cosolute; Rate: 0.5 °C/min; Concentration: 1.1 μM.

## 3. Discussion

The forces and the environmental conditions responsible for the stability of secondary or tertiary nucleic acids structures have been studied extensively. However, apart from the Watson–Crick duplex, few examples of secondary structures have demonstrated their existence in cell milieu. The total concentration of macromolecules is high in the interior of all cells and nucleic acids have evolved to function in crowding conditions. These conditions have determined their structure and stability and have allowed an understanding of their biological role. Several efforts have been focused on the impact of crowding conditions in relevant structures. One approach to understanding the forces for the stability of nucleic acids is to examine the effect of organic solvents or cosolutes. The result of adding cosolvents such as primary alcohols to aqueous solutions is a decrease in the thermal stability of duplex DNA. However, in many cases an opposite effect is observed in other secondary structures such as quadruplexes and triplexes. These results indicate the importance of secondary structures *in vivo*. In this paper, we have studied triplex stability with a variety of small cosolutes to simulate crowding environments with different hydrating properties. EtOH is considered to greatly reduce the water activity and acts as a good dehydrating and H-bonding agent, whereas ACN is a non-hydrogen-bonding dehydrating agent. In addition, DMSO may also act as a good dehydrating and H-formation agent.

The results based on spectroscopic methodologies (CD, molecular absorption and NMR) confirm that the triplex is formed preferentially with RNA TFO. The triplex stability is nearly maintained when the TFO is RNA in simulated crowding conditions. However, the DNA triplexes are strongly destabilized by cosolutes. The efficiency of stability changes by cosolute strongly depends on the cosolute nature. Triplex stability was more pronounced in EtOH. The water-releasing reaction favored by dehydrating solvents seems to drive the principal force in the formation of Hoogsteen base pair in the major groove of a DNA duplex.

The use of PEG cosolutes to simulate crowding conditions has been used in several studies [33] but recently is being questioned due to their possible interaction with macromolecules [51]. Larger molecules such as proteins or polysaccharides generate a significant crowding effect at a lower concentration. Nevertheless, these agents could not be studied by denaturing experiments as they loose their properties with the temperature.

On the other hand, kinetics of the triplex formation provided data in which the addition of RNA TFO to the hairpin was slower than DNA TFO in the presence of a cosolute. The thermal stability of the triplex is increased with the salt concentration. The addition of ethanol at 20% *w*/*v* decreases the dielectric response of the solutions and depending on salt concentration, the triplex is formed only in the case that TFO is RNA.

Polypurine-polypyrimidine sequences are especially abundant in promoter regions [52]. This high abundance of triplex-forming sequences has captured the attention of researchers, since it suggests the existence of a potential gene regulation mechanism based on a triplex formation [52]. The discovery of small non-coding RNA with regulatory properties such as miRNA has triggered the hypothesis of gene regulation mechanisms by miRNA [53,54,55,56]. The present work confirms the potential of small RNA molecules to act as triplex-forming compounds in molecular crowding conditions.

## 4. Experimental Section

### 4.1. Oligonucleotide Synthesis and Purification

The oligonucleotides prepared for this study (Table 1) were synthesized on an automated RNA/DNA synthesizer using β-cyanoethylphosphoramidite chemistry and following standard protocols. Hexaethylenglycol phosphoramidite (Glen Research) was used as a linker between the two strands of the hairpin. RNA TFO was prepared using 2′-*O*-TBDMS protected phosphoramidites (A^Bz^, G^dmf^, C^Ac^ and U). The TBDMS was removed using TBAF in THF. The oligonucleotides were purified by HPLC and characterized by mass spectrometry (Table S3).

### 4.2. UV Absorbance and Circular Dichroism Measurements

Oligonucleotides at 1.1 µM were re-suspended in 10 mM phosphate buffer and 100 mM NaCl pH 6.0 with the corresponding percentage (*w*/*v*) of cosolute and were then annealed at 85 °C. The solutions were slowly cooled to room temperature and left at 4 °C for one night. The thermal curves were carried out following the absorption change at 260 nm from 15 to 85 °C with a linear temperature ramp of 0.5 °C/min on a JASCO V-650 spectrophotometer equipped with a Peltier temperature control.

Thermodynamic parameters were calculated from UV-melting experiments as described elsewhere [39] using a Matlab program (R2009b version, Math-Works, Natick, MA, USA). This analysis assumes simple two-state equilibrium between the folded and unfolded states. The melting curves of hairpin and triplex structures were fitted using intramolecular and intermolecular (non-self-complementray) transitions, respectively.

The CD spectra were recorded on a JASCO spectropolarimeter J-810. Spectra were registered at 15 °C over a range of 205–320 nm with a scanning speed of 100 nm/min, a response time of 4 s, 0.5 nm data pitch and 1nm bandwidth. The samples (1 µM) were dissolved in the above buffers annealed, then slowly cooled to room temperature and left at 4 °C at least one night.

### 4.3. NMR Spectroscopy

Oligonucleotides at 0.3 mM were re-suspended in 10 mM Na-phosphate buffer and 100 mM NaCl pH 6.0 (H_2_O/D_2_O 9:1) with the corresponding percentage (*w*/*v*) of deuterated cosolute and were annealed at 85 °C, then slowly cooled to room temperature and left at 4 °C for one night.

The NMR spectra were recorded on a Bruker AV600 spectrometer operating at a frequency of 600.10 MHz for ^1^H. In addition, ^1^H spectra were recorded at a variable temperature ranging from 5 to 65 °C. ^1^H chemical shifts (δ) were measured in ppm and referenced to external DSS (2,2-dimethyl-2-silapentane-5-sulfonate sodium salt) set at 0.00 ppm.

### 4.4. TFO Association Analysis by Absorbance Decay

The formation of triplex structures by hairpin and the TFOs is accompanied by a decrease in UV absorbance. Kinetics of association is estimated from the decay curve associated to a triplex formation using the UV absorbance mixing method [43]. The measurements were carried out at 15 °C by mixing an equimolar amount of TFO and hairpin (1.1 μM) in 10 mM sodium phosphate buffer 100 mM NaCl with or without cosolvent. UV measurements were taken every 20 s. The decay curves were fitted by means of second order kinetics using a Matlab program (R2009b version, Math-Works, Natick, MA, USA).

## 5. Conclusions

We have demonstrated that molecular crowding conditions simulated with small cosolutes could be a key factor in the formation of triplex structures. In addition, the effect of the cosolutes discriminates and favors the formation of a triplex with RNA TFO. A fine tuning of several factors including crowding could boost triplex stability and consequently the biological applications of this structure. RNA oligonucleotides have resulted in ideal molecules for triplex formation and introduces a new concept of their biological role in gene regulation.

## Supplementary Materials

Supplementary materials can be found at http://www.mdpi.com/1422-0067/17/2/211/s1.

## References

[1] Bochman M.L., Katrin Paeschke K., Zakian V.A. (2012). DNA secondary structures: stability and function of G-quadruplex structures. Nat. Rev. Genet..

[2] Sainia N., Zhanga Y., Usdinb K., Lobacheva K.S. (2013). When secondary comes first—The importance of non-canonical DNA structures. Biochimie.

[3] Chan P.P., Glazer P.M. (1997). Triplex DNA: Fundamentals, advances, and potential applications for gene therapy. J. Mol. Med..

[4] Praseuth D., Guieysse A.L., Hélène C. (1999). Triple helix formation and the antigene strategy for sequence-specific control of gene expression. Biochim. Biophys. Acta.

[5] Mirkin S.M., Lyamichev V.I., Drushlyak K.N., Dobrynin V.M., Filippov S.A., Frank-Kamenetskii M.D. (1987). DNA H form requires a homopurine-homopyrimidine mirror repeat. Nature.

[6] LeDoan T., Perrouault L., Praseuth D., Habhoub N., Decout J.L., Thuong N.T., L’homme J., Hélène C. (1987). Sequence-specific recognition, photocrosslinking and cleavage of the DNA double helix by an oligo-(α)-thymidylate covalently linked to an azidoproflavine derivative. Nucleic Acids Res..

[7] Moser H.E., Dervan P.B. (1987). Sequence-specific cleavage of double helical DNA by triple helix formation. Science.

[8] Vasquez K.M., Wilson J.H. (1998). Triplex-directed modification of genes and gene activity. Trends Biochem. Sci..

[9] Vasquez K.M., Glazer P.M. (2002). Triplex-forming oligonucleotides: principles and applications. Q. Rev. Biophys..

[10] Radhakrishnan I., Patel D.J. (1994). DNA triplexes: Solution structures, hydration sites, energetics, interactions, and function. Biochemistry.

[11] Roberts R.W., Crothers D.M. (1992). Stability and properties of double and triple helices: Dramatic effects of RNA or DNA backbone composition. Science.

[12] Escudé C., Sun J.S., Rougée M., Garestier T., Hélène C. (1992). Stable triple helices are formed upon binding of RNA oligonucleotides and their 2′-*O*-methyl derivatives to double-helical DNA. C. R. Acad. Sci. III.

[13] Escudé C., Francois J.C., Sun J.S., Ott G., Sprinzl M., Garestier T., Hélène C. (1993). Stability of triple helices containing RNA and DNA strands: experimental and molecular modeling studies. Nucleic Acids Res..

[14] Han H., Dervan P.B. (1993). Sequence-specific recognition of double helical RNA and RNA.DNA by triple helix formation. Proc. Natl. Acad. Sci. USA.

[15] Gotfredsen C.H., Schultze P., Feigon J. (1998). Solution structure of an intramolecular pyrimidine-purine-pyrimidine triplex containing an RNA third strand. J. Am. Chem. Soc..

[16] Nakano S., Miyoshi D., Sugimoto N. (2014). Effects of molecular crowding on the structures, interactions, and functions of nucleic acids. Chem. Rev..

[17] Tateishi-Karimta H., Sugimoto N. (2014). Control of stability and structure of nucleic acids using cosolutes. Methods.

[18] Miyoshi D., Sugimoto N. (2008). Molecular crowding effects on structure and stability of DNA. Biochimie.

[19] Petraccone L., Pagano B., Giancola C. (2012). Studying the effect of crowding and dehydration on DNA G-quadruplexes. Methods.

[20] Albergo D.D., Turner D.H. (1981). Solvent effects on the thermodynamics of double-helix formation in (dG-dC)3. Biochemistry.

[21] Hickey D.R., Turner D.H. (1985). Solvent effects on the stability of A_7_U_7_p. Biochemistry.

[22] Nakano S., Yamaguchi D., Tateishi-Karimata H., Miyoshi D., Sugimoto N. (2012). Hydration changes upon DNA folding studied by osmotic stress experiments. Biophys. J..

[23] Spink C.H., Chaires J.B. (1999). Effects of hydration, ion release, and excluded volume on the melting of triplex and duplex DNA. Biochemistry.

[24] Nakano S., Karimata H., Ohmichi T., Kawakami J., Sugimoto N. (2004). The effect of molecular crowding with nucleotide length and cosolute structure on DNA duplex stability. J. Am. Chem. Soc..

[25] Rozners E., Moulder J. (2004). Hydration of short DNA, RNA and 2′-OMe oligonucleotides determined by osmotic stressing. Nucleic Acids Res..

[26] Miyoshi D., Nakamura K., Tateishi-Karimata H., Ohmichi T., Sugimoto N. (2009). Hydration of Watson-Crick base pairs and dehydration of Hoogsteen base pairs inducing structural polymorphism under molecular crowding conditions. J. Am. Chem. Soc..

[27] Collie G.W., Parkinson G.N. (2011). The application of DNA and RNA G-quadruplexes to therapeutic medicines. Chem. Soc. Rev..

[28] Heddi B., Phan A.T. (2011). Structure of human telomeric DNA in crowded solution. J. Am. Chem. Soc..

[29] Vorlíčková M., Bednářová K., Kejnovská I., Kypr J. (2007). Intramolecular and intermolecular guanine quadruplexes of DNA in aqueous salt and ethanol solutions. Biopolymers.

[30] Jiang H.X., Cui Y., Zhao T., Fu H.W., Koirala D., Punnoose J.A., Kong D.M., Mao H. (2015). Divalent cations and molecular crowding buffers stabilize G-triplex at physiologically relevant temperatures. Sci. Rep..

[31] Nagatoishi S., Isono N., Tsumoto K., Sugimoto N. (2011). Hydration is required in DNA G-quadruplex-protein binding. ChemBioChem.

[32] Spink C.H., Chaires J.B. (1995). Selective stabilization of triplex DNA by Poly(ethylene glycols). J. Am. Chem. Soc..

[33] Goobes R., Cohen O., Minsky A. (2002). Unique condensation patterns of triplex DNA: Physical aspects and physiological implications. Nucleic Acids Res..

[34] Goobes R., Minsky A. (2001). Thermodynamic aspects of triplex DNA formation in crowded environments. J. Am. Chem. Soc..

[35] Mamajanov I., Engelhart A.E., Bean H.B., Hud N.V. (2010). DNA and RNA in anhydrous media: Duplex, triplex, and G-quadruplex secondary structures in a deep eutectic solvent. Angew. Chem. Int. Ed..

[36] Schwarz-Finsterle J., Stein S., Grossmann C., Schmitt E., Trakhtenbrot L., Rechavi G., Amariglio N., Cremer C., Hasmann M. (2007). Comparison of triple helical COMBO-FISH and standard FISH by means of quantitative microscopic image analysis. J. Biochem. Biophys Methods.

[37] Wang S., Friedman A.E., Kool E.T. (1995). Origins of high sequence selectivity: A stopped-flow kinetics study of DNA/RNA hybridization by duplex- and triplex-forming oligonucleotides. Biochemistry.

[38] Bernal-Méndez E., Leumann C.J. (2002). Stability and kinetics of nucleic acid triplexes with chimaeric DNA/RNA third strands. Biochemistry.

[39] Puglisi J.D., Tinoco I. (1989). Absorbance melting curves of RNA. Methods Enzymol..

[40] Gowers D.M., Fox K.R. (1999). Towards mixed sequence recognition by triple helix formation. Nucleic Acids Res..

[41] Sugimoto N., Wu P., Hara H., Kawamoto Y. (2001). pH and cation effects on the properties of parallel pyrimidine motif DNA triplexes. Biochemistry.

[42] Liu K., Miles H.T., Frazier J., Sasisekharan V. (1993). A novel DNA duplex. A parallel-stranded DNA helix with Hoogsteen base pairing. Biochemistry.

[43] Xodo L.E. (1995). Kinetic analysis of triple-helix formation by pyrimidine oligodeoxynucleotides and duplex DNA. Eur. J. Biochem..

[44] Torigoe H., Ryuji Shimizume R., Sarai A., Shindo H. (1999). Triplex formation of chemically modified homopyrimidine oligonucleotides: Thermodynamic and kinetic studies. Biochemistry.

[45] Rougée M., Faucon B., Mergny J.L., Barcelo F., Giovannangeli C., Garestier T., Hélène C. (1992). Kinetics and thermodynamics of triple-helix formation: Effects of ionic strength and mismatched. Biochemistry.

[46] Hari Y., Ijitsu S., Akabane-Nakata M., Yoshida T., Obika S. (2014). Kinetic study of the binding of triplex-forming oligonucleotides containing partial cationic modifications to double-stranded DNA. Bioorg. Med. Chem. Lett..

[47] Puri N., Majumdar A., Cuenoud B., Miller P.S., Seidman M.M. (2004). Importance of clustered 2′-*O*-(2-aminoethyl) residues for the gene targeting activity of triple helix-forming oligonucleotides. Biochemistry.

[48] Cheng A.J., van Dyke M.W. (1993). Monovalent cation effects on intermolecular purine-purine-pyrimidine triple-helix formation. Nucleic Acids Res..

[49] Sekharudua C.Y., Yathindraa N., Sundaralingama M. (1993). Molecular dynamics investigations of DNA triple helical models: Unique features of the Watson-Crick duplex. J. Biomol. Struct. Dyn..

[50] Alberti P., Arimondo P.P., Mergny J.L., Garestier T., Hélène C., Sun J.S. (2002). A directional nucleation-zipping mechanism for triple helix formation. Nucleic Acids Res..

[51] Hansel R., Lohr F., Foldynova-Trantirkova S., Bamberg E., Trantirek L., Dotsch V. (2011). The parallel G-quadruplex structure of vertebrate telomeric repeat sequences is not the preferred folding topology under physiological conditions. Nucleic Acids Res..

[52] Goñi J.R., de la Cruz X., Orozco M. (2004). Triplex-forming oligonucleotide target sequences in the human genome. Nucleic Acids Res..

[53] Bagasra O., Stir A.E., Pirisi-Creek L., Creek K.E., Bagasra A.U., Glenn N., Lee J.S. (2006). Role of micro-RNAs in regulation of lentiviral latency and persistence. Appl. Immunohistochem. Mol. Morphol..

[54] Kanak M., Alseiari M., Balasubramanian P., Addanki K., Aggarwal M., Noorali S., Kalsum A., Mahalingam K., Pace G., Panasik N. (2010). Triplex-forming microRNAs form stable complexes with HIV-1 provirus and inhibit its replication. Appl. Immunohistochem. Mol. Morphol..

[55] Toscano-Garibay J.D., Aquino-Jarquin G. (2014). Transcriptional regulation mechanism mediated by miRNA-DNA·DNA triplex structure stabilized by Argonaute. Biochim. Biophys. Acta.

[56] Buske F.A., Mattick J.S., Bailey T.L. (2011). Potential *in vivo* roles of nucleic acid triple-helices. RNA Biol..

