# Supplementary material for: The Effect of Small Cosolutes that Mimic Molecular Crowding Conditions on the Stability of Triplexes Involving Duplex DNA
Source: Int J Mol Sci. 2016 Feb 5;17(2):211. doi: 10.3390/ijms17020211 (PMC4783943; doi:10.3390/ijms17020211)
Supplement: Supplementary file 1 [file ijms-17-00211-s001.pdf]

# Supplementary Materials: The Effect of Small Cosolutes that Mimic Molecular Crowding Conditions on the Stability of Triplexes Involving Duplex DNA

**Anna Aviñó, Stefania Mazzini, Raimundo Gargallo and Ramon Eritja**

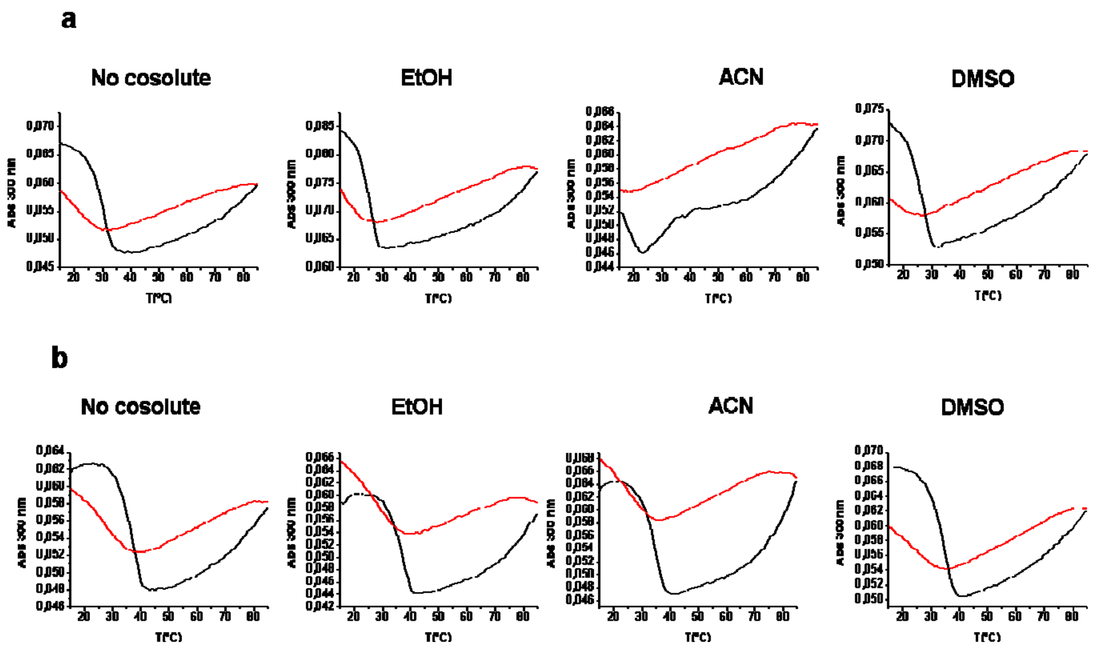


**Figure S1.** Melting (in black) and annealing (in red) curves of triplex formed with hairpin with (**a**) TFA DNA 2 (**b**) TFA RNA 2 in the presence of 20% (*v*/*w*) of cosolutes, 10 mM phosphate buffer 100 mM NaCl pH 6 at 295 nm.

**Table S1.** Melting temperatures ($T_m$, °C) [a] of the expected triplexes formed with hairpin 2 and TFA DNA 2 and TFO RNA 2 at 295 nm.

| $T_m$ (°C) | Expected Transition | No Cosolute | 20% EtOH | 20% ACN | 20% DMSO |
|---|---|---|---|---|---|
| Hairpin duplex 2 + DNA TFO 2 | Triplex to Duplex | 26.5 | 24.7 | 18.2 | 26.2 |
| Hairpin duplex 2 + RNA TFO 2 | Triplex to Duplex | 36.6 | 36.6 | 34.4 | 34.3 |

[a] 295 nm, 10 mM phosphate buffer 100mM NaCl pH 6 and the 20% *w*/*v* of cosolute; Heating rate: 0.5 °C/min.

**Table S2.** Apparent thermodynamic parameters of hairpin and the expected triplexes formed with hairpins 1 and 2 and the corresponding TFO DNAs and RNAs.

| **ΔH Kcal/mol** | **Expected Transition** | **No Cosolute** | **20% EtOH** | **20% ACN** | **20% DMSO** |
|---|---|---|---|---|---|
| Hairpin 1 | Duplex to SS | −69 ± 5 | −80 ± 1 | −78 ± 1 | −71 ± 3 |
| Hairpin 1 + TFO | Triplex to Duplex | −134 ± 19 | – | – | – |
| DNA 1 | Duplex to SS | −72 ± 3 | −79 ± 2 | −74 ± 1 | −69 ± 2 |
| Hairpin 1 + TFO | Triplex to Duplex | −150±15 | −130 ± 14 | −168 ± 24 | −116±7 |
| RNA 1 | Duplex to SS | −73 ± 4 | −80 ± 3 | −71 ± 2 | −73 ± 3 |
| Hairpin 2 | Duplex to SS | −77 ± 5 | −81 ± 6 | −71 ± 4 | −72 ± 6 |
| Hairpin 2 + TFO | Triplex to Duplex | −140 ± 3 | −168 ± 4 | −177 ± 5 | −144 ± 5 |
| DNA 2 | Duplex to SS | −93 ± 5 | −114 ± 2 | −99 ± 2 | −106 ± 3 |
| Hairpin 2 + TFO | Triplex to Duplex | −147 ± 4 | −130 ± 3 | −83 ± 4 | −109 ± 5 |
| RNA 2 | Duplex to SS | −112 ± 2 | −115 ± 3 | -102 ± 2 | −122 ± 2 |
| **ΔS° Kcal/mol** | **Expected Transition** | **No Cosolute** | **20% EtOH** | **20% ACN** | **20% DMSO** |
| Hairpin 1 | Duplex to SS | −205 ± 15 | −247 ± 3 | −239 ± 4 | −212 ± 9 |
| Hairpin 1 + TFO | Triplex to Duplex | −435 ± 66 | – | – | – |
| DNA 1 | Duplex to SS | −213 ± 9 | −241 ± 5 | −227 ± 3 | −207 ± 6 |
| Hairpin 1 + TFO | Triplex to Duplex | −487 ± 53 | −412 ± 47 | −547 ± 81 | −371±26 |
| RNA 1 | Duplex to SS | −215 ± 13 | −243 ± 7 | −213 ± 5 | −218 ± 9 |
| Hairpin 2 | Duplex to SS | −230 ± 16 | −249 ± 19 | −215 ± 12 | −214 ± 17 |
| Hairpin 2 + TFO | Triplex to Duplex | −428 ± 10 | −528 ± 13 | −568 ± 16 | −450 ± 15 |
| DNA 2 | Duplex to SS | −268 ± 15 | −343 ± 7 | −299 ± 5 | −313 ± 7 |
| Hairpin 2 + TFO | Triplex to Duplex | −446 ± 12 | −393 ± 9 | −244 ± 14 | −328 ± 18 |
| RNA 2 | Duplex to SS | −324 ± 5 | −343 ± 10 | −310 ± 4 | −361 ± 7 |
| **$\Delta G^{\circ}_{37}$ Kcal/mol** | **Expected Transition** | **No Cosolute** | **20% EtOH** | **20% ACN** | **20% DMSO** |
| Hairpin 1 | Duplex to SS | −6.3 | −3.4 | −4.0 | −5.2 |
| Hairpin 1 + TFO | Triplex to Duplex | 0.8 | – | – | – |
| DNA 1 | Duplex to SS | −6.5 | −4.6 | −3.5 | −5.0 |
| Hairpin 1 + TFO | Triplex to Duplex | 1.0 | −2.6 | 1.3 | −1.5 |
| RNA 1 | Duplex to SS | −6.9 | −4.7 | −3.4 | −5.2 |
| Hairpin 2 | Duplex to SS | −5.9 | −4.3 | −3.7 | −5.5 |
| Hairpin 2 + TFO | Triplex to Duplex | −7.2 | −3.7 | −0.6 | −4.3 |
| DNA 2 | Duplex to SS | −9.4 | −7.8 | −5.9 | −8.6 |
| Hairpin 2 + TFO | Triplex to Duplex | −9.2 | −8.9 | −7.5 | −7.6 |
| RNA 2 | Duplex to SS | −11.2 | −8.3 | −6.2 | −10.0 |

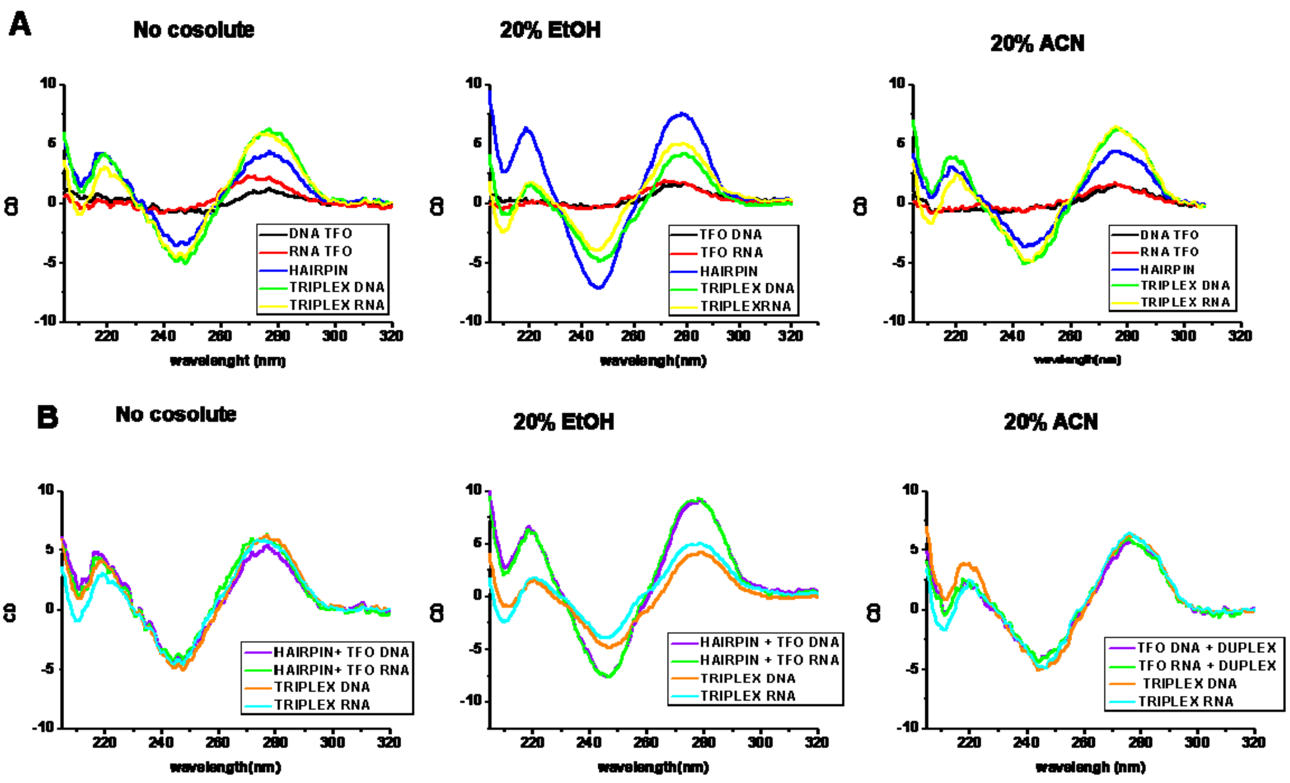


**Figure S2.** (**A**) CD spectra of TFOs, hairpin 1 and the triplexes formed by the addition TFOs to hairpin 1 without cosolute and in 20% EtOH and ACN; (**B**) Comparison of CD spectra of the arithmetic sum of CD spectra of hairpin 2 and TFOs and the CD spectra of the triplex structures. Buffer 10 mM phosphate buffer, 100 mM NaCl, pH 6.

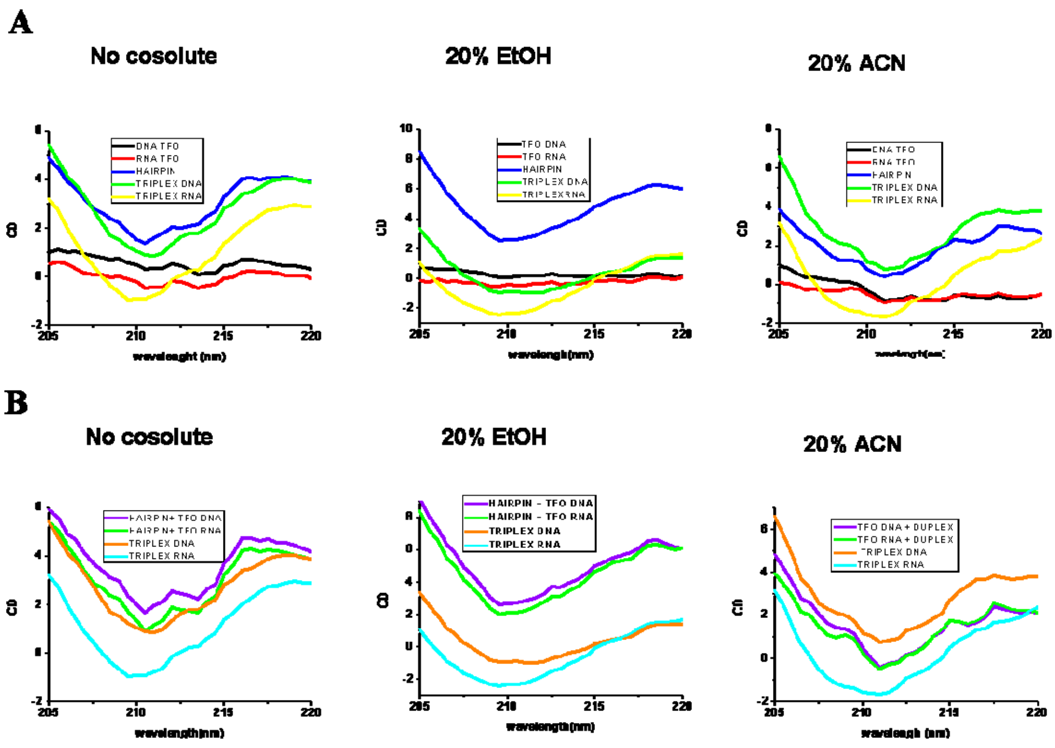


**Figure S3.** Amplification of the 205–220 nm region of CD spectra shown in Figure S2. (**A**) CD spectra of TFOs, hairpin 1 and the triplexes formed by the addition TFOs to hairpin 1; (**B**) Comparison of CD spectra of the arithmetic sum of CD spectra of hairpin 2 and TFOs and the CD spectra of the triplex structures.

**Table S3.** Oligonucleotide sequences and mass spectrometry data (MALDI) for the oligonucleotides used in this work.

| Name | Sequence | M (Expected) | M (Found) |
|---|---|---|---|
| Hairpin 1 | GAAAAGGAAGGA 5'<br>(EG)6<br>CTTTTCCTTCCT 3' | 7695.8 | 7691.3 |
| TFO DNA1 | 5′TCCTTCCTTTTC3′ | 3513.4 | 3514.3 |
| TFO RNA 1 | 5′UCCUUCCUUUUC3′ | 3607.4 | 3608.9 |
| Hairpin 2 | AGAAAAGAGGAAAGG 5'<br>(EG)6<br>TCTTTTCTCCTTTCC 3' | 9549.0 | 9549.8 |
| TFO DNA 2 | 5′CCTTTCCTCTTTTCT3′ | 4411.0 | 4413.9 |
| TFO RNA 2 | 5′CCUUUCCUCUUUUCU3′ | 4525 | 4528.4 |
